# Crop Disease Classification on Inadequate Low-Resolution Target Images

**DOI:** 10.3390/s20164601

**Published:** 2020-08-16

**Authors:** Juan Wen, Yangjing Shi, Xiaoshi Zhou, Yiming Xue

**Affiliations:** College of Information and Electrical Engineering, China Agricultural University, Beijing 100083, China; wenjuan@cau.edu.cn (J.W.); sy20183081433@cau.edu.cn (Y.S.); zhouxiaoshi0713@163.com (X.Z.)

**Keywords:** super-resolution, Generative Adversarial Networks, Convolutional Neural Networks, disease classification

## Abstract

Currently, various agricultural image classification tasks are carried out on high-resolution images. However, in some cases, we cannot get enough high-resolution images for classification, which significantly affects classification performance. In this paper, we design a crop disease classification network based on Enhanced Super-Resolution Generative adversarial networks (ESRGAN) when only an insufficient number of low-resolution target images are available. First, ESRGAN is used to recover super-resolution crop images from low-resolution images. Transfer learning is applied in model training to compensate for the lack of training samples. Then, we test the performance of the generated super-resolution images in crop disease classification task. Extensive experiments show that using the fine-tuned ESRGAN model can recover realistic crop information and improve the accuracy of crop disease classification, compared with the other four image super-resolution methods.

## 1. Introduction

Crop diseases are generally caused by the environment, soil, pests and pathogens. They pose a severe threat to the quality and security of agricultural production [1,2]. At the same time, crop diseases also cause losses to farmers. Taking prompt action could reduce losses. However, it is hard to detect the diseases in time through manual work.

With the development of computer science, it has become a hot topic to identify crop diseases based on computer vision and machine learning techniques. Earlier studies were based on feature extraction techniques. Alsuwaidi et al. [3] applied adaptive feature selection and ensemble learning for crop disease classification. Pantazi et al. [4] employed Local Binary Patterns (LBPs) for feature extraction and a one-class classifier to classify leaf diseases in various crop species. In recent years, image analysis methods based on deep learning have been used for crop disease identification and other purposes in agriculture, such as plant phenotypic analysis. Jia et al. [5] used transfer learning to classify tomato pests and diseases on leaf images based on VGG16 network. Zhang et al. [6] proposed global pooling dilated convolutional neural network (GPDCNN), which integrated the advantages of global pooling and dilated convolution to identify cucumber leaf diseases. Meanwhile, in order to construct a cost-effective system to diagnose diseases and symptoms of mango leaves, a multi-layer convolutional neural network (MCNN) [7] was proposed to classify mango leaves infected by anthracnose disease. It surpassed other approaches on a real-time dataset that includes 1070 images of the Mango tree leaves. Furthermore, based on the open dataset Plant Village [8], Too et al. [9] conducted a comparative study on the fine-tuned convolutional neural network (CNN) models for crop disease identification, including VGG16 [10], Inception V4 [11], ResNet with 50, 101 and 152 layers [12], and DenseNets [13] with 121 layers.

Since unmanned aerial vehicles (UAVs) have become increasingly popular in the agriculture industry in the past few years, some attempts have been made to identify crop diseases based on UAV images. Su et al. [14] collected UAV multispectral images by low-altitude UAVs and low-cost multispectral cameras. They then applied machine learning algorithms to monitor wheat yellow rust, making a significant contribution to yellow rust monitoring at farmland scales. Similar to their work, Cao et al. applied low-altitude remote sensing UAV images to detect Sclerotinia sclerotiorum on oilseed rape leaves [15]. Additionally, Kerkech et al. [16] utilized color information of UAV images to detect vine diseases based on a CNN model.

No matter what device was used to obtain the experimental images, one thing in common among these previous work was that high-resolution (HR) images were required for model training to ensure classification accuracy. In order to obtain HR images, high-quality cameras or sensors are required [17], which are costly and inefficient. In particular, if a UAV is used to capture HR images, it has to fly at a low altitude [14]. However, the drone propellers’ spinning motion will create turbulence and shake the leaves, which makes pictures blurry and unclear. According to Torres-Sánchez et al. [18], the ideal application scenario for UAVs is to fly at a high altitude to capture as many plants as possible. However, in such a case, the resolution of images will not be high enough for disease recognition. To solve this problem, Yamamoto et al. [19] first utilized the super-resolution (SR) method to transform low-resolution (LR) images to HR images for crop disease recognition. They applied a super-resolution convolution neural network (SRCNN) [20] to recover tomato leaf details and achieved better performance comparing with the results obtained from the original LR images. Cap et al. [21] used SRCNN and a Generative Adversarial Network (GAN) [22] to generate high-resolution images for detecting cucumber diseases, largely boosting the classification performance.

Because GAN has shown excellent ability in image SR tasks, in this article, we train a crop image super-resolution model based on GAN. Then we conduct crop disease classification on the generated SR images. Specifically, an enhanced super-resolution GAN (ESRGAN) [23] is trained to generate SR images on the Plant Village dataset [8], which is an open-source dataset with multiple plants and diseases. One major problem in our work is that it can be challenging to train a stable GAN model with insufficient labeled datasets. To address this issue, we use data augmentation to increase training samples. Furthermore, a base model pre-trained on ImageNet [24] is adopted to set the initial parameters of ESRGAN, and then transfer learning is applied to fine-tune the model twice in different learning rates to achieve a better quality of the SR images. Since tomato samples have more disease categories than other plants in the Plant Village dataset, tomato is chosen as the target crop in this paper. A VGG16 network is trained by transfer-learning and utilized to identify different types of tomato diseases, in order to verify the classification performance on the generated SR images. Extensive experiments are conducted to show the superiority of the proposed method compared with SRCNN and three conventional image scaling methods: bilinear, cubic, and lanczos4.

Our main contributions are mainly—(1) to handle low-resolution crop images, an ESRGAN model is built and trained to generate the HR images which are comparable to the original images. (2) To make the model work appropriately in case of inadequate crop data, we apply the transfer learning strategy to fine-tune the parameters of the ESRGAN in two separate steps. (3) Using the fine-tuned ESRGAN, which is one of the most potential SR algorithms, we can recover more realistic crop images and further improve the accuracy of crop disease classification.

The remainder of this article is as follows. Section 2 introduces the effective architecture of ESRGAN. Section 3 describes proposed method in details. Experimental details and results are covered in Section 4. Finally, the conclusion is provided in Section 5.

## 2. Related Work

### 2.1. Image Super-Resolution Methods

Image SR methods aim to recover detailed and spatial HR images from the corresponding LR images [25]. Recently, deep learning-based SR methods have become a persistent hot topic. SRCNN proposed by Dong et al. [20] established a mapping between low- and high-resolution images, which became a pioneer work of deep learning-based methods. After that, different network architectures and other strategies were put forward to improve the SR performance, mainly evaluated by Peak Signal-to-Noise Ratio (PSNR) [26,27,28,29,30,31,32]. In recent years, Shamsolmoali et al. introduced a progressive dilated convolution network which used progressive dilated densely connections and nonlinear learnable activation function to obtain complex features. Consequently, the network achieved satisfying performance in image SR tasks with few layers [33]. Yamamoto et al. [19] applied SRCNN to recover SR tomato leaf images and showed that the accuracy obtained on SR images was better by a large margin than those on LR images. However, images reconstructed via PSNR-oriented approaches can only capture limited perceptually relevant differences, that is, higher PSNR does not necessarily reflect a better perceptual result [34].

To improve the visual quality of SR images, some researchers proposed perceptual-driven methods. Perceptual loss [35] was applied to optimize SR model in feature space rather than pixel space. Furthermore, some researchers introduced GAN to generate SR images resembling realistic images. One of the milestones of GAN-based methods was SRGAN [34], which was constructed by residual blocks [12] and optimized with perceptual loss. Experiments showed that SRGAN significantly enhanced the visual quality of reconstruction over the PSNR-oriented methods. Based on SRGAN, Wang et al. proposed ESRGAN [23]. They improved the generator by designing the Residual-in-Residual Dense Block (RRDB), which had high capacity and low training complexity. Moreover, they improved the discriminator by utilizing Relativistic average GAN (RaGAN) [36]. Benefit from the adversarial structure and perceptual-driven SR strategies, ESRGAN can generate SR images with excellent visual effect.

GAN-based SR models are used in various image SR tasks. In scene recognition tasks, Wang et al. [37] proposed a text-attentional Conditional Generative Adversarial Network (CGAN) for text image SR in natural scene. The proposed model introduced effective channel and spatial attention mechanisms to enhance the original CGAN. It performed well on the public text image dataset. In handwriting recognition tasks, an end-to-end trainable framework was proposed by jointing GAN, deep back projection network (DBPN), and bidirectional long short term memory (BLSTM) [38]. The framework achieved state-of-the-art performances on both printed and handwritten document enhancement and recognition. In object recognition tasks, Xi et al. [39] proposed a Representation Learning Generative Adversarial Network (RLGAN) to generate SR image representation for tiny object recognition. RLGAN significantly improved the classification results on the challenging task of LR object recognition.

### 2.2. Transfer Learning

At present, more and more machine learning application scenarios have appeared. The existing supervised methods with better performance require a large amount of labeled data. Labeling data is a tedious and costly task. As one of the solutions, transfer learning has attracted more and more attention. Recently, many transfer learning approaches have emerged. Chen et al. [40] proposed a novel subspace alignment method for domain adaptation (DA). The method generated source subspace close to the target subspace by re-weighting the source samples. To match the source domain and target domain, data transformation and mapping are often used. In Reference [41], Xiao et al. proposed a projection-based feature transformation method for feature adaption between source and target domain.

In classification tasks, transfer learning allows us to learn a general classifier using a large amount of labeled data from the source domain and a small amount of labeled data from the target domain. A robust information-theoretic transfer learning framework was proposed in Reference [42] for classifier adaptation. The framework compensated for the loss of generalization performance caused by insufficient data through prior knowledge modeling. Furthermore, a novel deep transfer learning (DTL) model was proposed by applying sparse auto-encoder (SAE) and the maximum mean discrepancy term (MMDT) [43]. SAE extracted raw data features, and MMDT minimized the discrepancy penalty between training and testing data. The prediction accuracy of DTL on the famous motor bearing dataset was as high as 99.82%. Based on transfer learning, it is easier to achieve domain-invariant representation and domain transformation for GANs. A novel transfer learning framework with GAN architecture was proposed in Reference [44]. The model contains three parts: an encoder, a generator, and a duplex adversarial discriminators. It achieved state-of-the-art performance on unsupervised domain adaptation of digital classification and target recognition.

## 3. Materials and Methods

### 3.1. Proposed Method

**Figure 1:**
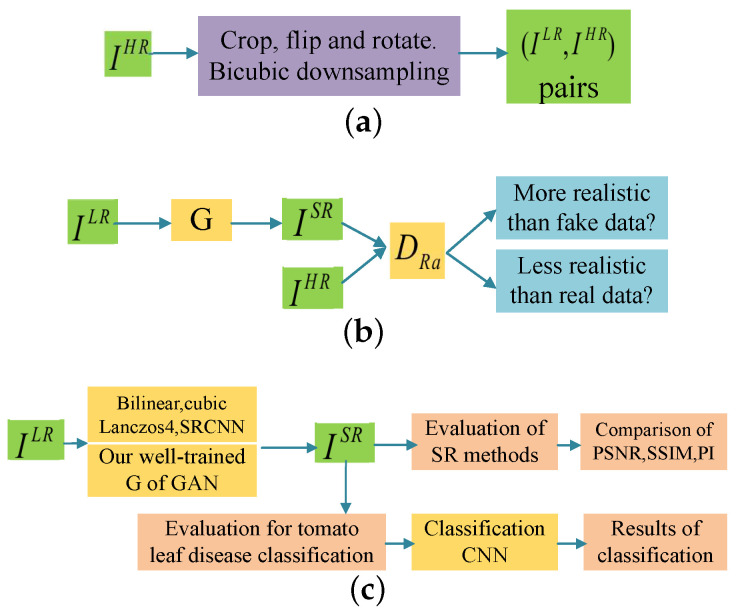
The three steps of our work. (**a**–**c**) represent the process of data processing, Generative Adversarial Network (GAN) model training and model evaluation, respectively.

In this paper, our task is to conduct crop disease classification based on inadequate low-resolution target images. To ensure the classification performance, we apply image super-resolution methods to transform the low-resolution crop images into HR images, trying to see how the performance can be improved by using these HR images instead. ESRGAN is chosen in our experiments due to its powerful ability in image SR tasks. Like most GAN-based models, ESRGAN can easily lead to non-convergence or over-fitting under insufficient data. One of the biggest challenges of our work is that there are not enough crop images to train our ESRGAN. In this paper, data augmentation and transfer learning are used to train ESRGAN under insufficient target images. First, we apply a basic model pre-trained on a public dataset ImageNet [24], which contains 1000 different classes. Then, the model parameters are fine-tuned with small-scale target images from the Plant Village dataset [8] to improve SR performance. Figure 1 shows the three-step process of our work.

(1) Data processing: as shown in Figure 1a, to build the classification model, it is necessary to prepare the LR and HR image pairs for model training. Images from the Plant Village dataset can be considered as HR images because these images themselves are of high quality. So we denote the cropped images with size of 128×128 pixels from Plant Village dataset as $I^{HR}$. Then $I^{HR}$ are flipped and rotated to enlarge the number of training samples. We obtain the HR images by bicubic interpolation with downsampling factor r=4. In this way, $I^{HR}$ can be converted to LR image $I^{LR}$ and the pair $\left(I^{LR},I^{HR}\right)$ can be used as the training sample of our GAN model.

(2) Model training: the process is shown in Figure 1b. Firstly we get a pre-trained generator *G* of ESRGAN, which is trained on ImageNet and saved as RRDB_ESRGAN_x4.pth, available on the website: https://github.com/xinntao/ESRGAN. Then we fine-tune this ESRGAN model using the crop dataset. We iteratively train the generator and the discriminator with adversarial training strategy. We end up with a well-trained *G*, which can be used to transfer the LR target images into HR ones. Details can be seen in Section 4.2.

(3) Evaluation: the evaluation is depicted in Figure 1c. Four other SR methods will be used for comparison. We first evaluate the quality of generated images $I^{SR}$ by PSNR, structural similarity index (SSIM) [45], and perceptual index (PI) [46]. Then the classification results based on VGG16 [10] through different SR methods are compared and analyzed.

### 3.2. Network Architecture

Our model adopts the training strategy of the original GAN, which optimizes the generator and discriminator in an alternating manner. The task of the generator *G* is to fool the discriminator by generating SR images similar to HR images. Conversely, the discriminator (denoted as $D_{Ra}$) is trained to distinguish the generated images from the real ones. In contrast to PSNR-oriented SR methods, ESRGAN applies perceptual loss in *G* to get natural and high-quality images.

**Figure 2:**
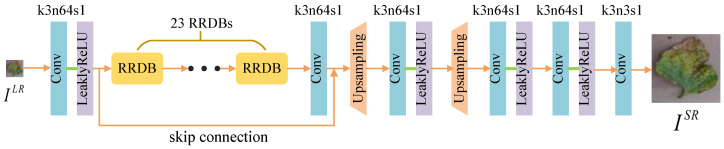
Architecture of generator network *G*. In each convolutional layer, *k*, *n*, and *s* represent kernel size, number of feature maps, and stride.

**Figure 3:**
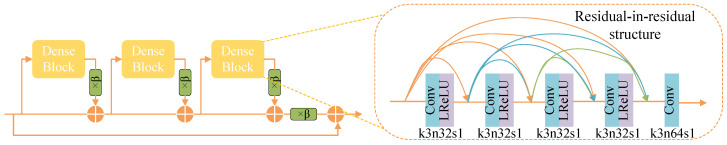
Residual-in-Residual Dense Block (RRDB) with residual scaling parameter β. In each convolutional layer, *k*, *n*, and *s* represent kernel size, number of feature maps, and stride.

**Figure 4:**
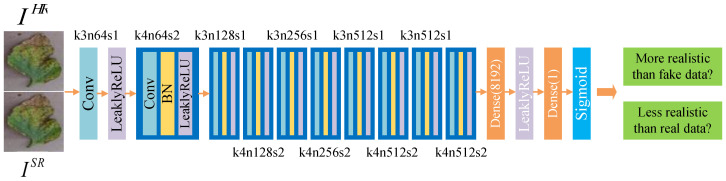
Architecture of discriminator network $D_{Ra}$. In each convolutional layer, *k*, *n*, and *s* represent kernel size, number of feature maps, and stride.

(1) The Generator: The generator is depicted in Figure 2. The input LR image $I^{LR}$ is fed to a convolutional layer with 3×3 filter kernels followed by LeaklyReLU as the activation function. 23 RRDBs, each of which is composed of dense blocks [13] and a multi-level residual network with five convolutional layers, are connected to the first convolutional layer [12] (See in Figure 3). In general, the RRDBs can magnify network capacity. Another convolutional layer with 3×3 kernels and 64 feature maps is added after the RRDB group to integrate features and match the data dimension. The scale factors of two upsampling layers are set to 2 to achieve image SR for 4× upscaling factors. Other convolutional layers are the same as the first one except that the final convolutional layer has three feature maps.

(2) The Discriminator: The discriminator is based on RaGAN [36]. It learns to determine which of the two input images is more realistic. The architecture of $D_{Ra}$ is depicted in Figure 4. It contains ten convolutional layers with 3×3 and 4×4 filter kernels appearing in an alternating way. Specifically, the kernel size *k*, the number of feature maps *n*, and stride *s* in each convolutional layer are showed in Figure 4. Batch-normalization (BN) layers [47] are connected behind convolutional layers to counteract the internal co-variate shift. $I^{HR}$ denotes the real HR crop image, and $I^{SR}$ is the fake HR image generated by the generator from the LR image $I^{LR}$. $I^{HR}$ has the same size as $I^{SR}$. Two dense layers and a final sigmoid activation function are used to predict the probability that an original real image $I^{HR}$ is relatively more realistic than a generated fake image $I^{SR}$.

(3) Loss Functions: $D_{Ra}$ has two outputs, denoted by $D_{real}$ and $D_{fake}$, respectively. $D_{real}$ is the average probability that the predicted result of the discriminator is an original HR image, and $D_{fake}$ is the average probability that the predicted result of the discriminator is the generated SR image. They can be expressed as Equations (1) and (2).

(1) $$D_{real}=C\left(I^{HR}\right)-E\left(C\left(I^{SR}\right)\right)$$

(2) $$D_{fake}=C\left(I^{SR}\right)-E\left(C\left(I^{HR}\right)\right),$$

where C(I) means discriminator output. E(·) means taking the average in the mini-batch data.

The loss of the discriminator $D_{Ra}$ is denoted by $L_{D}^{Ra}$. It can be divided into two parts: $L_{D_{real}^{Ra}}$ and $L_{D_{fake}^{Ra}}$. Formulas of $L_{D}^{Ra}$, $L_{D_{real}^{Ra}}$ and $L_{D_{fake}^{Ra}}$ can be expressed as Equations (3)–(5), respectively.

(3) $$L_{D}^{Ra}=L_{D_{real}^{Ra}}+L_{D_{fake}^{Ra}}$$

(4) $$L_{D_{real}^{Ra}}=-E_{I^{HR}}\left[\log\left(D_{Ra}\left(I^{HR},I^{SR}\right)\right)\right]$$

(5) $$L_{D_{fake}^{Ra}}=-E_{I^{SR}}\left[\log\left(1-D_{Ra}\left(I^{SR},I^{HR}\right)\right)\right],$$

where $D_{Ra}\left(I^{HR},I^{SR}\right)=\sigma \left(C\left(I^{HR}\right)-E_{I^{SR}}\left[C\left(I^{SR}\right)\right]\right)$, σ means sigmoid function.

The adversarial loss for generator *G* can be expressed as a symmetrical form as Equation (6).

(6) $$\begin{array}{cc} L_{G}^{Ra}= & -E_{I^{HR}}\left[\log\left(1-D_{Ra}\left(I^{HR},I^{SR}\right)\right)\right]\\ \; & -E_{I^{SR}}\left[\log\left(D_{Ra}\left(I^{SR},I^{HR}\right)\right)\right] \end{array}$$

Furthermore, the total loss of *G* is shown in Equation (7):

(7) $$L_{G}=L_{perceptual}+\alpha L_{G}^{Ra}+\beta L_{1},$$

where $L_{1}=E_{I^{SR}}\left|\right|I^{SR}-I^{HR}{\left|\right|}_{1}$ is the content loss which is used to evaluate the 1-norm distance between the recovered image $I^{SR}$ and the ground-truth $I^{HR}$. $L_{G}^{Ra}$ is an adversarial loss for generator, and we choose SR-MINC loss [46] as an appropriate perceptual loss $L_{perceptual}$, which is based on a fine-tuned VGG model for objection recognition and focuses on textures instead of object [48]. α, β are the coefficients to balance different loss terms.

### 3.3. Datasets and Metrics

The crop disease images used in our experiments are obtained from Plant Village dataset [8], which includes 54,309 images of 14 kinds of crops, such as tomato, corn, grape, apple, and soybean (available at: https://github.com/spMohanty/PlantVillage-Dataset/tree/master/raw/color). Since tomato is one of the most produced crops and has the largest number of diseases in the Plant Village dataset, it is chosen as the target crop in this paper. The size of each image in Plant Village is 256×256 pixels (denoted as original HR images). The number of tomato images is up to 18,160 in this dataset. There are 9 kinds of tomato disease classes, as well as the healthy class, shown in Table 1.

PSNR and SSIM [45] are two common metrics for evaluating the quality of images. They are frequently used to evaluate SR algorithms. PSNR between two images *f* and *g* with m×n pixels is defined as below Equation (8). A higher PSNR indicates better quality of generated images.

(8) $$PSNR=10\cdot \log_{10}\left(\frac{255^{2}}{MSE}\right)$$

where

(9) $$MSE=\frac{1}{mn}\sum_{i=0}^{m-1}\sum_{j=0}^{n-1}{\left[f\left(i,j\right)-g\left(i,j\right)\right]}^{2}.$$

And SSIM is calculated in Equation (10). Higher value of SSIM indicates better image quality.

(10) $$SSIM=\frac{\left(2\mu_{x}\mu_{y}+C_{1}\right)\left(2\sigma_{xy}+C_{2}\right)}{\left(\mu_{x}^{2}\mu_{y}^{2}+C_{1}\right)\left(\sigma_{x}^{2}+\sigma_{y}^{2}+C_{2}\right)},$$

where *x* and *y* represent the 7×7 windows in image *f* and *g*, $\mu_{x}$ and $\mu_{y}$ represent the average value of *x* and *y*, $\sigma_{x}^{2}$ and $\sigma_{y}^{2}$ represent the variance of *x* and *y*, and $\sigma_{xy}$ represents the covariance of *x* and *y*. $C_{1}$ and $C_{2}$ are variables to stabilize the division with weak denominators. Since we use RGB multi-channel images, these indices are calculated for each channel and then the average values of the channels are calculated.

However, several studies indicate that PSNR and SSIM cannot thoroughly evaluate perceptual-driven SR methods, such as SRGAN [34] and ESRGAN [23]. For this reason, Ledig et al. [34] proposed the mean opinion score (MOS) testing. In addition, Wang et al. [23] suggested applying PI in PIRM-SR Challenge [46] as an evaluation metric (more details in https://www.pirm2018.org/PIRM-SR.html). To better measure model performance, we also use PI for quantitative evaluation. Calculation of PI value depends on Ma’s score [49] and NIQE [50]. The expression is shown below in Equation (11). A lower PI value represents better perceptual quality. In other words, the image is more real and natural. We use the MATLAB program provided by sponsors of the competition to calculate PI values.

(11) $$PI=\frac{1}{2}\left(\left(10-Ma\right)+NIQE\right).$$

### 3.4. Crop Disease Classification

Since VGG16 [10] is a standard and straightforward image classification model, which performs well in the balance between training time and classification accuracy, it is chosen as the classifier in our experiments. We apply the classic VGG16 model, which consists of 13 convolution layers and 3 dense layers. The size of input and output layers of VGG16 is variable and adaptable. When the size of the input images changes, we need to change the setting of the width and height of the input layer of VGG16. In other words, the width and height of the input layer should be equal to the width and height of the input images. Similarly, the number of output classes should be equal to the number of neurons of the output layer. Specifically, if we perform a 6-class classification experiment with image size 64×64 pixels, the width and height of the input layer should be set to 64, and the number of neurons of the output layer should be set to 6. If we perform a 10-class classification experiment with image size 128×128 pixels, the width and height of the input layer are modified to 128, and the number of neurons in the output layer is modified to 10. Each layer is followed by ReLU activation function, which increases the non-linearity. Moreover, the MaxPooling layers are added to the second, fourth, seventh, tenth, and twelfth convolutional layers to reduce the dimension. Small filters with size 3×3 are applied to reduce the numbers of parameters and improve computational efficiency. Meanwhile, we fine-tune the VGG16 classification models trained on ImageNet with the Plant Village dataset, to achieve better classification performance and save computing resources.

## 4. Experiments

### 4.1. Experiment Setup

Most computations are conducted using python 3.5 on Ubuntu 16.04 system in our experiments. We implement the models with the PyTorch framework (version 1.1.0) and train them using a NVIDIA GeForce GTX 1070 GPU. A small part of image processing and PI calculation are carried out by MATLAB 2018a. We divide 18160 tomato leaf images from Plant Village database as training, validation, and testing sets, accounting for 80%, 10%, and 10%, respectively. All experiments apply a scaling factor of ×4 between LR and HR images. The size of the original HR images is 256×256 pixels. Since a larger patch size requires more computing resources and training time, the cropped HR patch size is 128×128 pixels. Furthermore, cropped HR images are flipped and rotated for data augmentation. Since GPU memory is an issue, the batch size is set to 16. In future work, we will consider accumulating gradients across batches to optimize the training process and improve efficiency. SRCNN [20] consists of three convolutional layers, and the size of the kernel is 9×9, 1×1, and 5×5. Mean-square error (MSE) is used as the loss function of the model. We trained SRCNN on the Plant Village dataset for comparison.

### 4.2. Train with Transfer Learning

**Figure 5:**
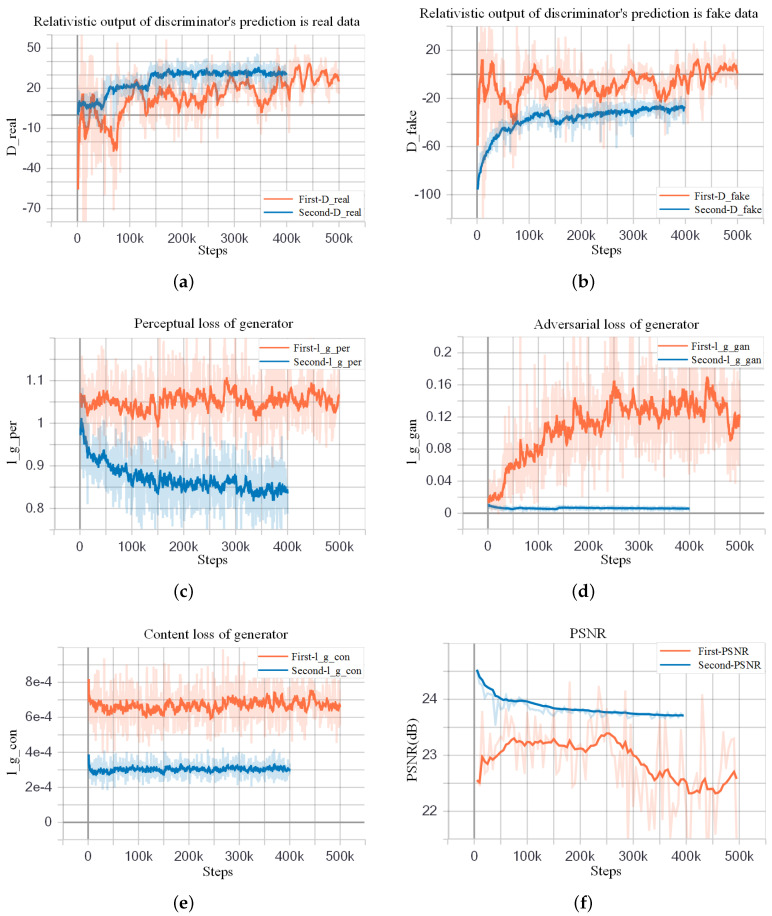
Comparison results of the first and second fine-tune training. In (**a**,**b**), The blue curves are smoother and have a larger mean absolute value of the difference between $D_{real}$ and $D_{fake}$ than orange ones, which indicates better training of $D_{Ra}$ in second fine-tuning process. The sudden change of the discriminator output at 50k and 125k should be caused by the changes in the learning rate. In (**c**–**e**), blue curves are smoother with smaller absolute losses. In (**f**), compared to the first fine-tuning, the PSNR of the second fine-tuning is higher, which also reflects the good performance of the second fine-tuning.

We use a pre-trained ESRGAN model provided by Wang et al. [23] to initialize the parameters for better quality and faster convergence (available on: https://github.com/xinntao/ESRGAN). This model is trained on ImageNet and does not work well in crop images. However, Wang only released the pre-trained generator *G* (denoted as $G_{pre}$) and did not release the pre-trained discriminator *D*. We fine-tune our model twice to compare the training performance in different training conditions. In the first fine-tuning, we use the pre-trained generator model $G_{pre}$ as the initialization of our *G* and initialize $D_{Ra}$ randomly. This causes an imbalance between the abilities of $D_{Ra}$ and *G*. In other words, *G*’s generation ability is strong, and $D_{Ra}$’s discriminative ability is poor. When the first fine-tuning finished, we got the trained *G* (denoted as $G_{1}$) and the trained $D_{Ra}$ (denoted as $D_{Ra1}$).The turbulent orange training curves in Figure 5 indicates insufficient training of the first fine-tuning step. So we consider carrying out the second fine-tune training with different hyperparameters settings. In the second fine-tuning, we utilize $G_{1}$ model and $D_{Ra1}$ as initialization of *G* and $D_{Ra}$. Because $G_{1}$ and $D_{Ra1}$ have learned certain feature distribution, the discriminator becomes more powerful, and the abilities of *G* and $D_{Ra}$ become relatively balanced. Thus, we get the $G_{2}$ and $D_{Ra2}$ at the end of the second fine-tuning.

To be specific, in the first fine-tuning step, we train the generator *G* using the loss function in Equation (7) with $\alpha =5\times 10^{-3}$ and $\beta =1\times 10^{-2}$, where learning rate is set to $1\times 10^{-4}$ and halved at [50k,100k,200k,300k,400k] iterations (learning rate decay factor γ=0.5). The learning rate setting for discriminator is the same as the generator. We use Adam [51] with $\beta_{1}=0.9$ and $\beta_{2}=0.99$ as the optimizer of generator and discriminator. The maximum number of iterations is set to 500k, and checkpoint is saved every 5k steps (Settings are referred to Reference [23]). It took about six days for the first fine-tuning. In the second fine-tuning process, we used the trained model $G_{1}$ as initialization for *G* and the corresponding $D_{Ra1}$ as initialization for $D_{Ra}$. The learning rate of *G* and $D_{Ra}$ is set to $5\times 10^{-5}$, which is smaller than previous settings. Moreover, the learning rate is adjusted dynamically to help the model converge. The learning rate is halved at [50k,125k,200k,300k] iterations. Loss function coefficients are also modified: $\alpha =1\times 10^{-4}$ and $\beta =5\times 10^{-3}$. These settings emphasize the perceptual loss term. The maximum number of iterations is set to 400k. Other settings remain unchanged. It took around five days for second fine-tuning.

Furthermore, since BN layers are removed to make training stable, training such a deep network becomes a problem. When the weights are updated, the distribution of the inputs in deep layers may change after each mini-batch, making the algorithm difficult to converge. To solve this problem, we use residual scaling strategy [11], which scales down the residuals by multiplying a constant between 0 and 1 before adding them to the main path to prevent instability. Using smaller initialization parameters in the residual structure can make training easier to converge.

The comparison of two fine-tuning steps is shown in Figure 5. The orange curves show the first fine-tuning process, and the blue ones show the second fine-tuning process. We can see that the blue curves are smoother than the orange curves, revealing that the second fine-tuning is more stable and reliable. Figure 5a,b represent the two average relativistic output of $D_{Ra}$: $D_{real}$ and $D_{fake}$. In the second fine-tuning process, the value of $D_{real}$ and $D_{fake}$ finally stabilized at 30 and −30, respectively. And this indicates good training of $D_{Ra}$. l_g_per, l_g_gan, and l_g_con in Figure 5c–e, represent perceptual loss, adversarial loss, and content loss of *G*, respectively. It can be seen that the loss of the second training has decreased. PSNR is one of the metrics for evaluating SR methods. As shown in Figure 5f, compared to the first fine-tuning, the PSNR of the second fine-tuning is higher, which also reflects the good performance of the second fine-tuning. However, we can see that in the second training step, the average PSNR gradually decreases as the number of iterations increases. That is because the optimization goal of perceptual-driven SR methods is to minimize perceptual loss instead of mean squared reconstruction error (MSRE). This type of method sacrifices the PSNR performance in exchange for better image visual perception.

**Figure 6:**
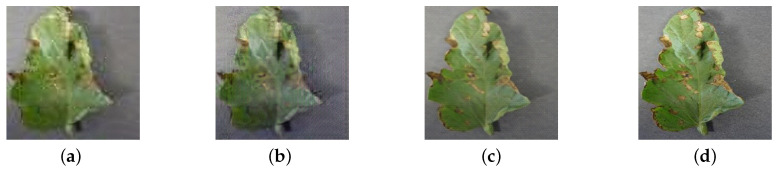
Visual comparison of super-resolution (SR) images generated from three training stages. (**a**) is from the pre-trained $G_{pre}$ based on ImageNet, (**b**) is from $G_{1}$ after the first fine-tuning and (**c**) is from $G_{2}$ after the second fine-tuning. (**d**) is the original high-resolution (HR) image.

An example of SR images generated by the pre-trained $G_{pre}$, first fine-tuned $G_{1}$, and the second fine-tuned $G_{2}$ can be seen in Figure 6. It can be observed that the image in Figure 6a only contains basic leaf shape and color information but lacks detailed information on lesions. After the first fine-tuning process, the image in Figure 6b is clearer and has sharper edges. However, it still lacks detailed information due to the different initialization strategies for the generator and the discriminator. The generated SR image from $G_{2}$ is realistic and natural, as shown in Figure 6c.

### 4.3. Evaluation of the Generated SR Images

**Figure 7:**
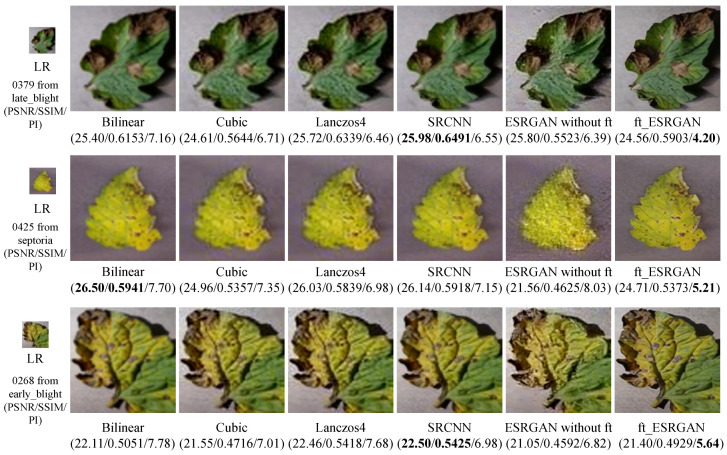
Examples of generated SR images. Our ft_ESRGAN produces sharper and more natural texture with richer visual information. “ESRGAN without ft” in the sixth column means ESRGAN without fine-tuning. And “ft_ESRGAN” in the seventh column means ESRGAN with second fine-tuning. [×4 upscaling].

To evaluate the quality of the generated SR images, we display some test image results in Figure 7, in which PSNR (evaluated on all RGB channels), SSIM, and PI (evaluation index for PRIM-SR Challenge) are compared. Among them, “ESRGAN without ft” in the sixth column means the results for ESRGAN without fine-tuning. It can be seen in Figure 7 that the PI values of three generated SR images by our second fine-tuned ESRGAN (denoted as ft_ESRGAN) are the lowest. However, their PNSR and SSIM values are not the highest. That is because, unlike these PSNR-oriented approaches, ESRGAN is mainly minimizing perceptual loss to enhance visual quality instead of minimizing MSRE. Besides, our ft_ESRGAN achieves better visual performance with more natural and authentic textures than the other four approaches.

We also calculate the average PSNR and SSIM of SR images generated by different SR methods from the test set (including 1812 images). The PI calculation is time-consuming, it takes about a minute to calculate PI value for one image. So we randomly choose 100 images from the test set (10 images are randomly chosen per category). The results are shown in Table 2. The average PNSR and SSIM of PSNR-oriented SRCNN are the highest, and the average PI of our perceptual-driven ft_ESRGAN is the lowest, which indicates that ft_ESRGAN could generate more realistic SR images with more comprehensive crop lesion details.

### 4.4. Classification Results

To verify whether the generated SR images by ft_ESRGAN contain rich information for classification, we conduct crop disease classification experiments on tomato leaves. Then we compare our model with the bilinear, cubic, lanczos4, and SRCNN. Considering the problem of data balance, we first choose 6 categories of tomato leaf images, each of which has a similar amount of samples. These 6 categories are bacterial spot (2027 images), late blight (1909), septoria leaf spot (1771), spider mites (1676), target spot (1404), and healthy (1591), respectively. The total number is 10,478 (see Table 1). Based on these original images, we conduct comparative experiments with different image sizes. By down-sampling HR images through bicubic kernel, we get two groups of LR images with 16×16 and 32×32 pixels. Then we reconstruct SR images using bilinear, cubic, lanczos4, SRCNN, and our ft_ESRGAN with a magnification scaling factor of ×4. After reconstruction, we generate two groups of SR images with 64×64 and 128×128 pixels for each SR method. We also show the classification results on HR and LR images as the upper and lower bounds of the experiment.

In these classification experiments, the image samples are randomly divided to form the training, validation, and testing sets with a ratio of 0.8, 0.1, and 0.1. We use a VGG16 model trained on ImageNet as the initialization for our classifier. We modify the setting of the width and height of the input layer and the number of output classes of the output layer to fit our image sizes of this 6-class classification task. Stochastic Gradient Descent (SGD) is used for optimization, and the learning rate is set to be $1\times 10^{-4}$. The maximum number of iterations is set to be $1\times 10^{4}$. The 6-class classification results on the test set are shown in Table 3.

From Table 3, we can see that the classification accuracies through SR images are much higher than the ones through LR images. The proposed ft_ESRGAN achieves the highest accuracies, reaching 93.59% and 95.60% for SR images with the sizes 64×64 and 128×128 pixels, respectively. Moreover, classification performance based on deep learning methods (SRCNN and ft_ESRGAN) is better than the conventional image scaling methods (Bilinear, Cubic, and Lanczos4).

To further evaluate the classification performance of the proposed model on an unbalanced dataset, we also conduct a comparative experiment in all 10 categories (see Table 1) using a similar process. The learning rate is set to $5\times 10^{-5}$, and the maximum of iterations is $1.5\times 10^{4}$. The number of neurons in the output layer is modified to 10. Other settings are the same as the 6-class classification experiments. The 10-class classification results are shown in Table 4.

From Table 4, it can be observed that the classification accuracies on SR images are much higher compared with those on LR images under both image sizes. Moreover, the classification performance on the generated SR images obtained by our ft_ESRGAN model is better than other methods. The above experiments show that the proposed ft_ESRGAN model can generate images with useful and specific information for classification tasks.

From Table 3 and Table 4, we can see that classification accuracy on LR images is the lowest. It reveals that LR images contain less useful information that can be captured by VGG16 for classification than SR or HR ones. Besides, because the size of the LR images is smaller than the size of SR and HR images, VGG16 may not be well trained for LR images due to its large amount of parameters, resulting in low classification accuracy. That is to say, VGG16 may not be a good tool for classifying the LR images. In this paper, the LR image accuracy is considered as a lower bound for classification, helping us to study the impact of SR methods for the classification tasks.

**Figure 8:**
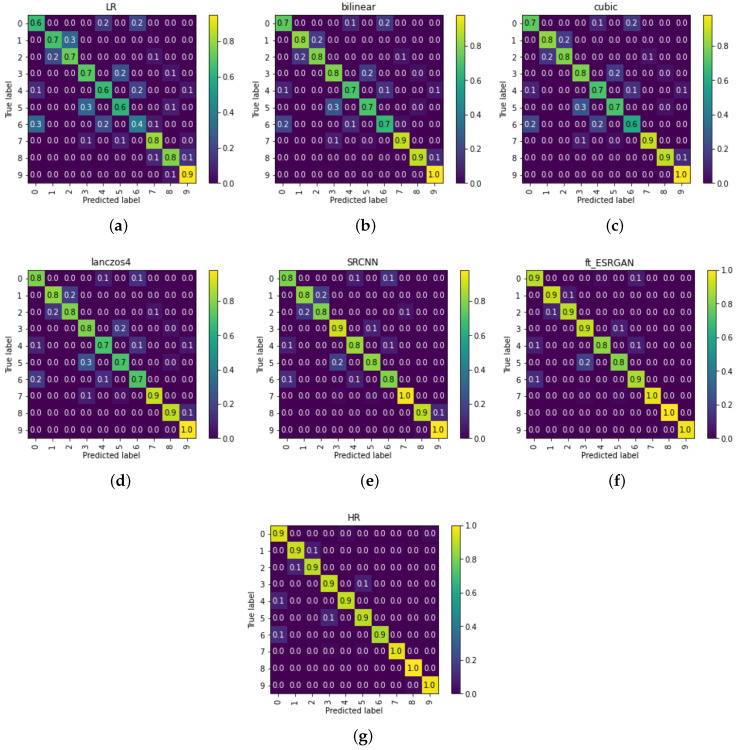
Confusion matrix of disease classification using using LR (32×32 pixels), SR (128×128 pixels) and HR images (128×128 pixels). Numbers on x and y axes indicate the ID of diseases in Table 1. (**a**) LR; (**b**) Bilinear; (**c**) Cubic; (**d**) Lanczos4; (**e**) SRCNN; (**f**) ft_ESRGAN; (**g**) HR.

To study the classification accuracy on each category, we show the confusion matrix for the second group of 10-class classification experiment (LR images: 32×32 pixels, SR and HR images: 128×128 pixels) in Figure 8. The results are normalized to 0–1 by the number of elements in each category. From Figure 8, We can see the classification accuracy gradually increases from LR to SR to HR. Among the chosen SR methods, our ft_ESRGAN performance is closest to the upper bound—the classification performance on HR images. Healthy class is the easiest category to identify. Furthermore, class 1 (early blight) and class 2 (late blight) are quite confounding. Similarly, class 0 (bacterial spot), class 4 (septoria leaf spot), and class 6 (target spot) are hard to distinguish from each other, too.

## 5. Conclusions

In this paper, we have proposed a method for crop disease identification on LR images by transferring LR images to SR images based on GAN. First, we employ ESRGAN on LR images to generate the corresponding SR images. Due to insufficient crop data, we apply transfer learning to fine-tune the model trained on ImageNet. After two fine-tuning steps, our SR model reaches a stable state, and the generated images achieve an excellent visual effect. Then we conduct disease classification experiments using the generated SR images. Experimental results show that the classification accuracy can be significantly improved by applying the proposed SR model, indicating that our SR model can reconstruct the useful information for identifying crop diseases. Due to the powerful reconstruction ability of ESRGAN, the performance achieved by the proposed model is better than those achieved by the other four methods. In our research, we utilized disease images taken by ground cameras rather than UAV cameras. Although our approach should be effective on UAV images, it is still necessary to verify our approach to images from UAV cameras for practical application in future works. Besides, The training efficiency and generalization ability of the model can be further improved. Furthermore, we can apply the SR model in object detection tasks. In this way, we can detect multiple diseases on one crop images. 

## Figures and Tables

**Table 1:** The number of each category tomato leaf images in Plant Village dataset.

No.	Name of Category	Number of Pictures
0	bacterial spot	2027
1	early blight	1000
2	late blight	1909
3	mold leaf	952
4	septoria leaf spot	1771
5	spider mites	1676
6	target spot	1404
7	tomato yellow curl virus	5357
8	tomato mosaic virus	373
9	healthy	1591

**Table 2:** The average Peak Signal-to-Noise Ratio (PSNR), structural similarity index (SSIM) and perceptual index (PI) of SR images generated by different SR methods.

Evaluation Index	Bilinear	Cubic	Lanczos4	SRCNN	ESRGAN without ft	ft_ESRGAN
PSNR(dB)	24.45	24.49	24.50	**25.42**	21.72	23.70
SSIM	0.4776	0.4710	0.4734	**0.5126**	0.3886	0.4678
PI	7.23	7.07	7.00	7.16	7.12	**6.10**

**Table 3:** Comparison of classification results for low-resolution (LR) and SR images based on 6 categories.

	Sample Size	Total Numbers	Accuracy
LR	16×16	1045	56.94%
bilinear	64×64	1045	88.42%
cubic	64×64	1045	88.61%
lanczos4	64×64	1045	89.36%
SRCNN	64×64	1045	92.54%
**ft_ESRGAN**	**64×64**	**1045**	**93.59%**
**HR**	**64×64**	**1045**	**95.41%**
LR	32×32	1045	80.48%
bilinear	128×128	1045	93.30%
cubic	128×128	1045	92.82%
lanczos4	128×128	1045	93.78%
SRCNN	128×128	1045	94.26%
**ft_ESRGAN**	**128×128**	**1045**	**95.60%**
**HR**	**128×128**	**1045**	**97.80%**

**Table 4:** Comparison of classification results for LR and SR images based on 10 categories.

	Sample Size	Total Numbers	Accuracy
LR	16×16	1812	52.65%
bilinear	64×64	1812	71.14%
cubic	64×64	1812	73.01%
lanczos4	64×64	1812	72.79%
SRCNN	64×64	1812	80.13%
**ft_ESRGAN**	**64×64**	**1812**	**85.38%**
**HR**	**64×64**	**1812**	**89.96%**
LR	32×32	1812	72.74%
bilinear	128×128	1812	82.40%
cubic	128×128	1812	81.02%
lanczos4	128×128	1812	83.44%
SRCNN	128×128	1812	86.09%
**ft_ESRGAN**	**128×128**	**1812**	**90.78%**
**HR**	**128×128**	**1812**	**95.14%**

## References

[1] Harvey C.A., Zo Lalaina R., Rao N.S., Radhika D., Hery R., Rivo Hasinandrianina R., Haingo R., Mackinnon J.L. (2014). Extreme vulnerability of smallholder farmers to agricultural risks and climate change in Madagascar. Philos. Trans. R. Soc. Lond. Ser. A.

[2] Tai A.P.K., Martin M.V., Heald C.L. (2014). Threat to future global food security from climate change and ozone air pollution. Nat. Clim. Chang..

[3] Alsuwaidi A., Grieve B., Yin H. (2018). Feature-Ensemble-Based Novelty Detection for Analyzing Plant Hyperspectral Datasets. IEEE J. STARS.

[4] Pantazi X., Moshou D., Tamouridou A.A. (2019). Automated leaf disease detection in different crop species through image features analysis and One Class Classifiers. Comput. Electron. Agric..

[5] Jia S., Jia P., Hu S., Liu H. Automatic detection of tomato diseases and pests based on leaf images. Proceedings of the 2017 Chinese Automation Congress (CAC).

[6] Zhang S., Zhang S., Zhang C., Wang X., Shi Y. (2019). Cucumber leaf disease identification with global pooling dilated convolutional neural network. Comput. Electron. Agric..

[7] Singh U.P., Chouhan S.S., Jain S., Jain S. (2019). Multilayer Convolution Neural Network for the Classification of Mango Leaves Infected by Anthracnose Disease. IEEE Access.

[8] Hughes D.P., Salathe M. (2015). An open access repository of images on plant health to enable the development of mobile disease diagnostics through machine learning and crowdsourcing. arXiv.

[9] Too E.C., Li Y., Njuki S., Liu Y. (2018). A comparative study of fine-tuning deep learning models for plant disease identification. Comput. Electron. Agric..

[10] Simonyan K., Zisserman A. (2014). Very Deep Convolutional Networks for Large-Scale Image Recognition. arXiv.

[11] Szegedy C., Ioffe S., Vanhoucke V., Alemi A. (2016). Inception-v4, Inception-ResNet and the Impact of Residual Connections on Learning. arXiv.

[12] He K., Zhang X., Ren S., Sun J. Deep Residual Learning for Image Recognition. Proceedings of the IEEE Conference on Computer Vision and Pattern Recognition (CVPR) 2016.

[13] Huang G., Liu Z., van der Maaten L., Weinberger K.Q. Densely Connected Convolutional Networks. Proceedings of the 2017 IEEE Conference on Computer Vision and Pattern Recognition (CVPR).

[14] Su J., Liu C., Coombes M., Hu X., Wang C., Xu X., Li Q., Guo L., Chen W.-H. (2018). Wheat yellow rust monitoring by learning from multispectral UAV aerial imagery. Comput. Electron. Agric..

[15] Cao F., Liu F., Guo H., Kong W., Zhang C., Yong H. (2018). Fast Detection of Sclerotinia Sclerotiorum on Oilseed Rape Leaves Using Low-Altitude Remote Sensing Technology. Sensors.

[16] Kerkech M., Hafiane A., Canals R. (2018). Deep learning approach with colorimetric spaces and vegetation indices for vine diseases detection in UAV images. Comput. Electron. Agric..

[17] Abdulridha J., Batuman O., Ampatzidis Y. (2019). UAV-Based Remote Sensing Technique to Detect Citrus Canker Disease Utilizing Hyperspectral Imaging and Machine Learning. Remote Sens..

[18] Torres-Sánchez J., López-Granados F., De Castro A., Peña-Barragán J.M. (2013). Configuration and Specifications of an Unmanned Aerial Vehicle (UAV) for Early Site Specific Weed Management. PLoS ONE.

[19] Yamamoto K., Togami T., Yamaguchi N. (2017). Super-Resolution of Plant Disease Images for the Acceleration of Image-based Phenotyping and Vigor Diagnosis in Agriculture. Sensors.

[20] Chao D., Chen C.L., He K., Tang X. Learning a Deep Convolutional Network for Image Super-resolution. Proceedings of the European Conference on Computer Vision 2014—ECCV 2014.

[21] Cap Q.H., Tani H., Uga H., Kagiwada S., Iyatomi H. Super-Resolution for Practical Automated Plant Disease Diagnosis System. Proceedings of the 2019 53rd Annual Conference on Information Sciences and Systems (CISS).

[22] Goodfellow I.J., Pouget-Abadie J., Mirza M., Bing X., Warde-Farley D., Ozair S., Courville A., Bengio Y. Generative Adversarial Nets. Proceedings of the Advances in Neural Information Processing Systems 27 (NIPS 2014).

[23] Wang X., Yu K., Wu S., Gu J., Liu Y., Dong C., Qiao Y., Loy C.C. ESRGAN: Enhanced Super-Resolution Generative Adversarial Networks. Proceedings of the European Conference on Computer Vision (ECCV) 2018.

[24] Deng J., Dong W., Socher R., Li L.J., Li K., Li F.F. ImageNet: A Large-Scale Hierarchical Image Database. Proceedings of the IEEE Conference on Computer Vision and Pattern Recognition (CVPR).

[25] Glasner D., Bagon S., Irani M. Super-resolution from a single image. Proceedings of the IEEE International Conference on Computer Vision (ICCV).

[26] Lai W.S., Huang J.B., Ahuja N., Yang M.H. Deep Laplacian Pyramid Networks for Fast and Accurate Super-Resolution. Proceedings of the IEEE Conference on Computer Vision and Pattern Recognition (CVPR).

[27] Kim J., Lee J.K., Lee K.M. Deeply-Recursive Convolutional Network for Image Super-Resolution. Proceedings of the IEEE Conference on Computer Vision and Pattern Recognition (CVPR).

[28] Tai Y., Yang J., Liu X. Image Super-Resolution via Deep Recursive Residual Network. Proceedings of the IEEE Computer Vision and Pattern Recognition (CVPR).

[29] Tai Y., Yang J., Liu X., Xu C. MemNet: A Persistent Memory Network for Image Restoration. Proceedings of the 2017 IEEE International Conference on Computer Vision (ICCV).

[30] Haris M., Shakhnarovich G., Ukita N. Deep Back-Projection Networks For Super-Resolution. Proceedings of the IEEE Conference on Computer Vision and Pattern Recognition (CVPR).

[31] Zhang Y., Tian Y., Kong Y., Zhong B., Fu Y. Residual Dense Network for Image Super-Resolution. Proceedings of the IEEE Conference on Computer Vision and Pattern Recognition (CVPR).

[32] Zhang Y., Li K., Li K., Wang L., Zhong B., Fu Y. Image Super-Resolution Using Very Deep Residual Channel Attention Networks. Proceedings of the European Conference on Computer Vision (ECCV).

[33] Pourya Shamsolmoali X.L., Wang R. (2019). Single image resolution enhancement by efficient dilated densely connected residual network. Signal Process. Image Commun..

[34] Ledig C., Theis L., Huszar F., Caballero J., Aitken A., Tejani A., Totz J., Wang Z., Shi W. Photo-Realistic Single Image Super-Resolution Using a Generative Adversarial Network. Proceedings of the IEEE Computer Vision and Pattern Recognition (CVPR).

[35] Johnson J., Alahi A., Li F.F. Perceptual losses for real-time style transfer and super-resolution. Proceedings of the European Conference on Computer Vision (ECCV).

[36] Jolicoeur-Martineau A. (2018). The relativistic discriminator: A key element missing from standard GAN. arXiv.

[37] Wang Y., Su F., Qian Y. Text-Attentional Conditional Generative Adversarial Network for Super-Resolution of Text Images. Proceedings of the 2019 IEEE International Conference on Multimedia and Expo (ICME).

[38] Ray A., Sharma M., Upadhyay A., Makwana M., Chaudhury S., Trivedi A., Singh A., Saini A. An End-to-End Trainable Framework for Joint Optimization of Document Enhancement and Recognition. Proceedings of the 2019 International Conference on Document Analysis and Recognition (ICDAR).

[39] Xi Y., Zheng J., Jia W., He X., Li H., Ren Z., Lam K.M. (2020). See Clearly in the Distance: Representation Learning GAN for Low Resolution Object Recognition. IEEE Access.

[40] Chen S., Zhou F., Liao Q. Visual domain adaptation using weighted subspace alignment. Proceedings of the 2016 Visual Communications and Image Processing (VCIP).

[41] Xiao M., Guo Y. (2015). Feature Space Independent Semi-Supervised Domain Adaptation via Kernel Matching. IEEE Trans. Pattern Anal. Mach. Intell..

[42] Ramachandran A., Gupta S., Rana S., Venkatesh S. Information-theoretic transfer learning framework for Bayesian optimisation. Proceedings of the 2018 European Conference on Machine Learning (ECML).

[43] Wen L., Gao L., Li X. (2017). A New Deep Transfer Learning Based on Sparse Auto-Encoder for Fault Diagnosis. IEEE Trans. Syst. Man Cybern. Syst..

[44] Hu L., Kan M., Shan S., Chen X. Duplex Generative Adversarial Network for Unsupervised Domain Adaptation. Proceedings of the IEEE Conference on Computer Vision and Pattern Recognition (CVPR).

[45] Wang Z., Bovik A.C., Sheikh H.R., Simoncelli E.P. (2004). Image quality assessment: From error visibility to structural similarity. IEEE Trans. Image Process..

[46] Blau Y., Mechrez R., Timofte R., Michaeli T., Zelnik-Manor L. 2018 PIRM Challenge on Perceptual Image Super-resolution. Proceedings of the European Conference on Computer Vision (ECCV).

[47] He K., Zhang X., Ren S., Sun J. Identity Mappings in Deep Residual Networks. Proceedings of the European Conference on Computer Vision (ECCV).

[48] Bell S., Upchurch P., Snavely N., Bala K. Material Recognition in the Wild with the Materials in Context Database. Proceedings of the IEEE Conference on Computer Vision and Pattern Recognition (CVPR).

[49] Ma C., Yang C.Y., Yang X., Yang M.H. (2016). Learning a No-Reference Quality Metric for Single-Image Super-Resolution. Comput. Vis. Image Underst..

[50] Mittal A., Soundararajan R., Bovik A. (2013). Making a “Completely Blind” Image Quality Analyzer. IEEE Signal Process Lett..

[51] Kingma D., Ba J. (2014). Adam: A Method for Stochastic Optimization. arXiv.

